# Bioinformatics-based construction of prognosis-related methylation prediction model for pancreatic cancer patients and its application value

**DOI:** 10.3389/fphar.2023.1086309

**Published:** 2023-03-09

**Authors:** Tiansheng Cao, Hongsheng Wu, Tengfei Ji

**Affiliations:** Department of Hepatobiliary Surgery, Affiliated Huadu Hospital, Southern Medical University (People’s Hospital of Huadu District), Guangzhou, China

**Keywords:** pancreatic cancer, methylation, prognosis, prediction model, drugs

## Abstract

**Objective:** Pancreatic adenocarcinoma (PAAD) is a highly malignant gastrointestinal tumor with almost similar morbidity and mortality. In this study, based on bioinformatics, we investigated the role of gene methylation in PAAD, evaluated relevant factors affecting patient prognosis, screened potential anti-cancer small molecule drugs, and constructed a prediction model to assess the prognosis of PAAD.

**Methods:** Clinical and genomic data of PAAD were collected from the Tumor Genome Atlas Project (TCGA) database and gene expression profiles were obtained from the GTEX database. Analysis of differentially methylated genes (DMGs) and significantly differentially expressed genes (DEGs) was performed on tumorous samples with KRAS wild-type and normal samples using the “limma” package and combined analysis. We selected factors significantly associated with survival from the significantly differentially methylated and expressed genes (DMEGs), and their fitting into a relatively streamlined prognostic model was validated separately from the internal training and test sets and the external ICGC database to show the robustness of the model.

**Results:** In the TCGA database, 2,630 DMGs were identified, with the largest gap between DMGs in the gene body and TSS200 region. 318 DEGs were screened, and the enrichment analysis of DMGs and DEGs was taken to intersect DMEGs, showing that the DMEGs were mainly related to Olfactory transduction, natural killer cell mediated cytotoxicity pathway, and Cytokine -cytokine receptor interaction. DMEGs were able to distinguish well between PAAD and paraneoplastic tissues. Through techniques such as drug database and molecular docking, we screened a total of 10 potential oncogenic small molecule compounds, among which felbamate was the most likely target drug for PAAD. We constructed a risk model through combining three DMEGs (S100P, LY6D, and WFDC13) with clinical factors significantly associated with prognosis, and confirmed the model robustness using external and internal validation.

**Conclusion:** The classification model based on DMEGs was able to accurately separate normal samples from tumor samples and find potential anti-PAAD drugs by performing gene-drug interactions on DrugBank.

## 1 Background

Pancreatic adenocarcinoma (PAAD) is the 14th most common cancer globally (Kocarnik et al., 2022), with an estimated 458,918 confirmed pancreatic cancer cases and 432,242 death cases each year all over the world (Ferlay et al., 2019). The incidence of PAAD varies widely by country, as Europe and North America showed the highest age-standardized incidence, which was the lowest in South-Central Asia and Africa (Ilic and Ilic, 2016). Incidence rate of PAAD is generally higher in developed countries compared to developing countries, with a standardized incidence rate of 4.9/100,000 and 3.6/100,000 for men and women, respectively. In the United States, 5-year survival rate of PAAD is 9.3%, and it is the fourth leading factor resulting in cancer-related mortality (Gandhi et al., 2018). Apart from smoking, diabetes, alcohol drinking, obesity, occupational exposure and genetic factors, PAAD is as well an epigenetic disease (Goral, 2015; Midha et al., 2016; Hu et al., 2021). Abnormal DNA methylation patterns are a common human tumorous feature (Kulis and Esteller, 2010). From precancerous lesions to PAAD, epigenetic changes play an important role in the multistage carcinogenesis (Xu et al., 2019).

Epigenetics are changes in gene expression but not in DNA sequence, and the major epigenetic alteration leading to PAAD progression is DNA methylation (Wang et al., 2016). To detect epigenetic abnormalities in PAAD, it is necessary to identify genome-wide patterns of DNA methylation. Nones et al. (2014) used high-density arrays to capture 167 untreated PAAD sample methylation and compared it with normal tissue adjacent to the cancerous one and identified 3,522 abnormally methylated genes. In addition, partial methylation of CDKN1C promoter CpG islands and reduced expression of protein products are observed when comparing PAAD precursor cells methylation expression to normal pancreatic duct epithelial cells (Sato et al., 2008). Basic studies have shown that in PAAD precursor cells, CDKN1C gene is under-expressed and there is reduced expression of protein products and partial methylation of CDKN1C promoter CpG islands. The above evidence supports that aberrant DNA hypo/hypomethylation occurs in PAAD precursor lesions leading to further progression to PAAD (13).

As research continues, aberrant methylation of DNA CpG islands has become a prominent feature of PAAD and a potential diagnostic marker and therapeutic target for PAAD. However, the results of clinical trials were disappointing, probably due to the low level of epigenetic specificity (Matsubayashi et al., 2006; Marabelle et al., 2020). Therefore, in order to use methylation as a future therapeutic tool for PAAD, an in-depth understanding of the methylation expression profile and supporting pathways of PAAD is needed. According to the mutation and gene expression profile data of PAAD patients and gene expression profiles of normal pancreas from GTEX, this study screened differentially methylated and expressed genes (DMEGs), and confirmed that methylation was a reliable prognostic marker for PAAD and a potential oncogenic drug target for PAAD.

## 2 Materials and methods

### 2.1 Acquisition of clinical data, gene expression profiles and data processing

Methylation data, clinical follow-up data, and gene expression profiles of PPAD came from TCGA (https://portal.gdc.cancer.gov/) by means of UCSC Xena. The gene expression profiles of normal pancreas samples were obtained from the GTEX (http://www.gtexportal.org/home/index.html) databases using UCSC Xena.

For sample data reliability, we set the following inclusion criteria (Kocarnik et al., 2022): only normal samples and primary PAAD samples were retained (Ferlay et al., 2019); PAAD samples with wild-type KRAS gene were retained (Ilic and Ilic, 2016); PAAD samples with complete clinical data were retained. A total of 182 samples were obtained from TCGA, including 178 tumor samples, 70 KRAS wild-type tumor samples and 4 normal samples. A total of 167 normal pancreas samples were obtained from the GTEX database. In order to homogenize the data, the “sva” R package was applied to remove the batch effect from the combined data of the two datasets, and a total of 19,593 protein-coding genes were retained by ENSG conversion of gene symbols using genecode V35.

### 2.2 Analysis of differentially methylated genes (DMGs)

The Illumina HumanMethylation450 BeadChip matrix contained 380,097 probes of around 99% (*n* = 26,081) of the RefSeq genes. For each probe, the raw gene methylation intensity was expressed as a beta value. To identify differentially methylated CpG sites (DMS), PAAD tumor samples were compared with paracancer samples using the “limma” R package (Ritchie et al., 2015). The Benjamini and Hochberg (BH) method adjusted *p*-value of each methylation site to FDR (false discovery rate) (Ghosh, 2012). Statistical thresholds were set for FDR <0.01 and |delta β-value|> 0.1.

The CpG locus to gene match files were downloaded from the Illumina website (https://www.illumina.com/). In different regions (TSS200, TSS1500, Gene body, 5′-UTR, 3′-UTR, transcription start site, integration region), the average β-values of genes were calculated with the correspondence. Using the “limma” R package, the differentially methylated regions were calculated, where FDR <0.01, delta β-values < -0.1 were the demethylated regions, FDR <0.01, delta *β* > 0.1 were hypermethylated regions.

### 2.3 Analysis of differentially expressed genes, differentially methylated genes and pathways

Differentially expressed genes (DEGs) were analyzed for normal and tumor samples in the TCGA-PAAD cohort using the “limma” R package, and *p* values were adjusted using the Benjamini and Hochberg (BH) method, where FDR >0.01 and log2FC > 2 were up-regulated genes, and FDR >0.01 and log2FC < −2 were down-regulated genes.

To identify the relationship between gene methylation and gene expression profiles, we took the intersection of differentially methylated genes and DEGs to obtain differentially methylated and expressed genes (DMEGs) and classified them into four groups: HyperDown, HyperUp, HypoDown, HypoUp (Table 1). Then, we used Gene Ontology (GO) functional enrichment analysis and the Kyoto Encyclopedia of Genes and Genomes (KEGG) database through the “clusterProfiler,” “org.Hs.eg.db,” “enrichplot” and “ggplot2” R software packages (Wu et al., 2021), and FDR <0.05 was used as the screening condition to perform enrichment analysis of DEGs to discover the main biological characteristics of DEGs and plot the bubble map.

**TABLE 1:** Criteria for grouping DMEGs.

Groups	Methylation cut-off	Expression cut-off
HypoUp	FDR <0.01 and delta β-value < −0.1	FDR <0.01 and log2FC > 2
HypoDown	FDR <0.01 and delta β-value < −0.1	FDR <0.01 and log2FC < -2
HyperUp	FDR< 0.01 and delta β-value >0.1	FDR <0.01 and log2FC > 2
HyperDown	FDR <0.01 and delta β-value >0.1	FDR <0.01 and log2FC < -2

FDR:false discovery rate; log2FC: log2 fold change.

### 2.4 Marker evaluation of PAAD methylation and expression profiles

DEGs were proposed as tumor markers for the diagnosis of PAAD, and 50% of the expression profile data of DMEGs and methylation data of DMEGs for PAAD were the training set and 50% as the test set. The training set data were analyzed by principal component analysis (PCA) with the “prcomp” R function (Luu et al., 2017) to clarify the eigenvector weights of the principal components and construct a diagnostic model of PAAD, which was plotted and visualized using the “ggplot2” R software package (Maag, 2018). Finally, to evaluate the diagnostic advantage of PCA model for PAAD, the receiver operating characteristic (ROC) curves of the PCA model were plotted by the “pROC” R software package and the area under curve (AUC) was calculated for the training set and test set (Robin et al., 2011), where AUC showed a low accuracy at 0.5–0.7, higher accuracy at 0.7–0.9, and high accuracy at AUC above 0.9.

### 2.5 The prediction of DMEGs and target drugs

The use of key genes as potential therapeutic targets is a cornerstone in the development of therapeutic agents for sepsis. We determined PAAD and drug proximity based on drug-target pairs from the drugbank database (https://go.drugbank.com/) and the Protein-Protein interaction (PPI) network (threshold score of 400). Here, given distance d (s,t) as the shortest path between node s and node t (where s ∈ S, PAAD-related genes; t ∈ T, drug target genes), D (degree of related gene set nodes in PPI), T (set of drug target genes), S (PAAD-related genes), and the calculation is as follow:

$$d\left(S,T\right)=\frac{1}{\left|T\right|}\underset{t\in T}{\sum }\min_{s\in S}\left(d\left(s,t\right)+\omega \right)$$ (1)

where *ω*, the weight of the target gene, was calculated as ω = -ln (D+1) if the target gene was a gene in the PAAD-related gene set, otherwise ω = 0.

Next, between these simulated drug targets and the key gene set, we calculated the distance d (S,R), and generated the simulated reference distributions after performing random repetitions for 10,000 times, at the same time we the observed distances corresponding to the actual were scored using the mean and standard deviation of the μd (S,R) and σd (S,R) reference distributions and converted into a normalized scoring, i.e., the proximity z.

$$z\left(S,T\right)=\frac{d\left(S,T\right)-\mu_{d\left(S,R\right)}}{\sigma_{d\left(S,R\right)}}$$ (2)

Finally, a gene set distance density score map was constructed by normalized distance scoring.

### 2.6 Molecular docking

A technique for designing drugs based on receptor features and the way that drug molecules interact with receptors is called molecular docking. In the realm of computer-aided drug development, it is a theoretical modeling technique that primarily investigates the interaction between molecules (such as ligands and receptors) and forecasts their binding mechanism and affinities. (Lohning et al., 2017; Saikia and Bordoloi, 2019). Autodock Vina software was used in molecular docking (Trott and Olson, 2010). To prepare input files, we applied AutoDockTools 1.5.6. The pdb file of the protein came from Protein Data Bank (Velankar et al., 2021) with PDB ID 6SUK. The Polar hydrogens were added to the solution after all water molecules, potassium ions, and protein B chains had been eliminated. The zinc ion’s charge was modified in the receptor protein’s PDBQT file to +2.0, and the grid’s coordinates in each XYZ direction were −19.5, 74.5, and 34.8 during molecular docking. The lengths were 20 in each XYZ direction. The Lamarckian approach was utilized to determine the ligand molecule’s strongest binding mode. The maximum number of output conformations was set to 10, the exhaustiveness was set to 8, and the allowable energy range was set to a maximum of 3 kcal/mol. With the aid of Pymol, the output maps were processed.

### 2.7 Dynamics simulation

In this study, the binding stability of the receptor-ligand complex was assessed by performing molecular dynamics simulations of 100 ns (Zhou et al., 2022) using the Gromacs2019 package. In the molecular dynamics simulations the CHARMm36 force field was employed. With the aid of the CHARMM Common Force Field (CGenFF) software, the str files for the ligands were acquired. The system was dissolved in TIP3P water molecules in a dodecahedral box. At a concentration of 0.154 M, sodium and chloride ions were introduced to the system to neutralize its charge. Using a cutoff of 5,000 steps and the steepest descent algorithm, the solventized system’s energy was minimized. The LINCS method was used to restrict the length of covalent bonds. Using the PME technique, the total electrostatic interactions were determined. At constant temperature (300 K) and pressure (1 bar), NVT and NPT simulations were then run for 100 ps, with the compound’s confined atoms re-establishing the system’s equilibrium at its initial coordinates. Finally, a 100 ns long Pruduct MD run with a 2 fs time step was completed. The Gromacs built-in tool was used to determine the ligands’ root mean square deviation (RMSD) values.

### 2.8 Development and verification of the prognostic gene signature model associated with DMEG

In the TCGA-PAAD dataset, we first randomly and equally divided 241 KRAS wild samples into training (Train) and validation (Test) groups according to the ratio of 1:1, and then reduced the associated genes (Tibshirani, 1997) by Least absolute shrinkage and selection operator (Lasso) regression method. In regression analysis, by compressing some coefficients at the same time setting some coefficients to zero, Lasso regression can better solve multicollinearity. We choose the number of factors when the coefficients of independent variables tended to zero with the gradual increase of lambda. Then, we used the AIC deficit pool information criterion through stepwise regression that takes the statistical fit of the model and parameter numbers into account. A better model of smaller value indicated a sufficient fit of the model with fewer number of parameters (Zhang, 2016). After that, the “survminer” package was used to find the best cutoff (Niu et al., 2021) of the gene signature in the Train dataset of TCGA, and the PAAD was divided into two groups based on the cutoff value, and finally the log-rank test was used to compare the survival differences between the two groups.

To verify the robustness of the gene signature model, we first used the same model and the same coefficients as the training set in the validation set, and then compared the survival differences between the two groups by log-rank test. After that, we downloaded the expression profiles of PAAD from the ICGC database as well as clinical information, and then used the model constructed above to calculate each score separately and obtain the best cutoff, and then performed the survival curve analysis in the external dataset for the high- and low-risk groups.

### 2.9 Statistical analysis

All statistical analyses were operated in R software (version 4.1.2, https://cran.r-project.org/doc/manuals/R-lang.html). The optimal threshold of gene expression or score was selected for risk grouping of PAAD using the surv_cutpoint function of the “survminer” package. The Kaplan-Meier assessment method was used to assess the survival differences between the low-risk and high-risk groups, and the Log-rank test was used for comparison. Unless otherwise stated, all statistical tests were two-sided and *p* < 0.05 was considered statistically significant. Comparisons between multiple groups were performed and plotted using the “ggpubr” and “ggplot2” packages, and the statistical significance of box plots was assessed using the Mann-WhitneyU or Kruskal–Wallis tests.

## 3 Results

### 3.1 Analysis of differentially methylated genes in PAAD

**FIGURE 1:**
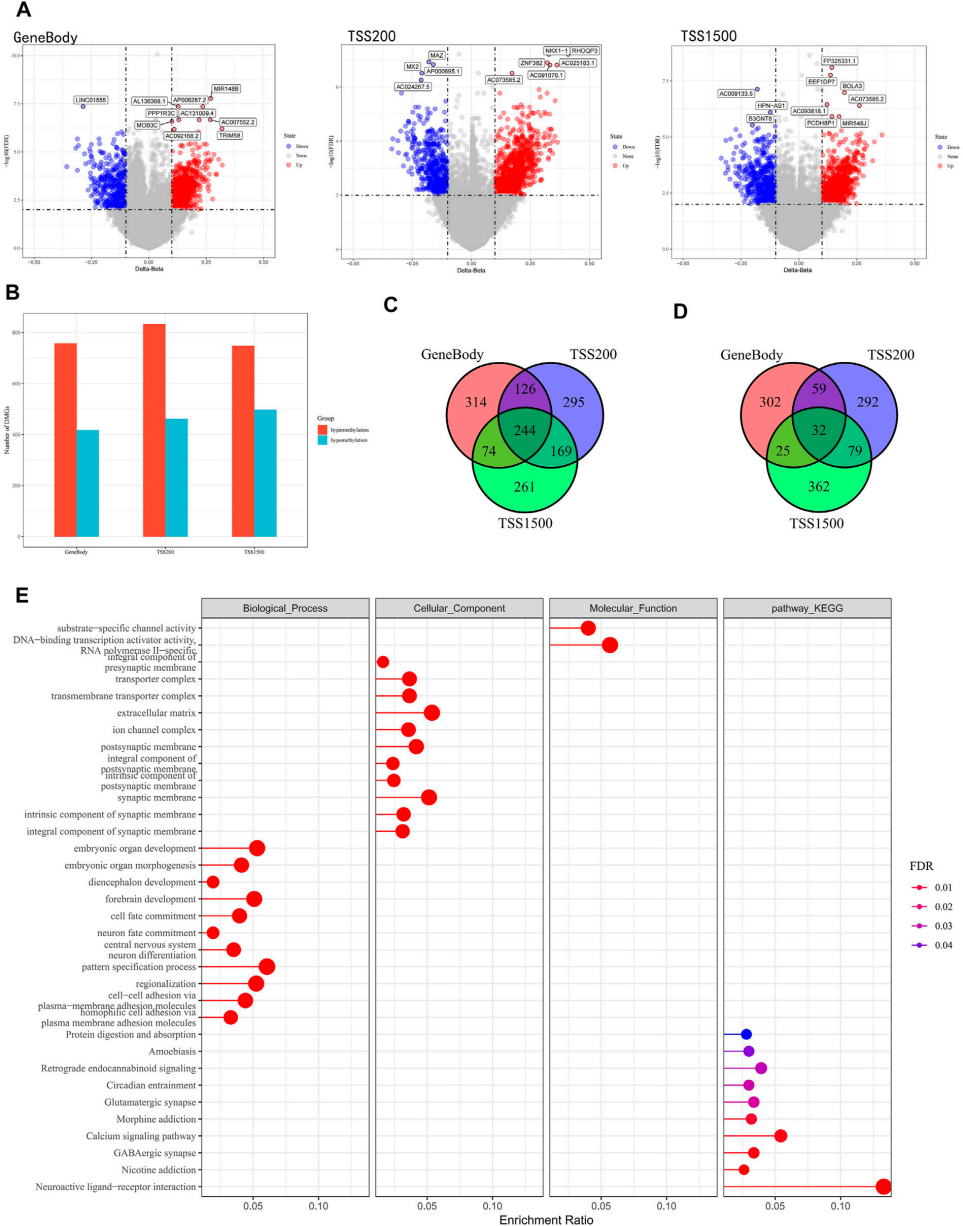
Analysis of PAAD differentially methylated genes. **(A)** Volcano plot of differentially methylated within the gene body, TSS200 and TSS1500 regions. **(B)** Histograms of differentially methylated genes within the three regions. **(C)** Venn diagram of hypermethylation within three different regions. **(D)** Venn diagrams of demethylated genes within three different regions. **(E)** KEGG and GO functional enrichment analysis of differentially methylated genes, where the color from blue to red indicates that the FDR is from large to small, and the dots from small to large represent the increasing number of enriched genes, left: hypermethylation, right: demethylation.

To identify differential gene methylation in PAAD, we first performed a comparative analysis of methylation data from 185 KRAS wild-type PAAD samples and 10 normal samples, and identified a total of 2,630 differentially methylated genes (FDR <0.01, |delta β-values| > 0.1, Figure 1A), within the Gene body region, 758 genes were hypermethylated and 418 genes were demethylated. 834 genes were hypermethylated and 462 genes were demethylated in TSS20; 748 genes were hypermethylated and 498 genes were demethylated in TSS1500. We found that the number of hypermethylation in the three regions was slightly larger than that of hypermethylation overall (Figure 1B). In the Gene body and TSS200 regions, the difference between hypermethylation and demethylation was the largest, with a ratio of about 1.8:1. Among the hypermethylated genes, 244 genes appeared in all three regions of Gene body, TSS20 and TSS1500, 369 genes appeared in two of them, and the remaining 870 genes appeared in only one region (Figure 1C). Among the demethylated genes, only 32 genes appeared in all three regions, and 163 genes appeared in two of them. These differentially methylated genes were mainly associated with GABAergic synapse, Neuroactive ligand-receptor interaction, Nicotine addiction, and other pathways, as shown by GO and KEGG functional enrichment analysis (Figure 1D). (Figure 1E). The above results confirmed that PAAD methylation was region-specific.

### 3.2 Analysis of differential genes in PAAD and combined analysis of differentially metaylated genes

**FIGURE 2:**
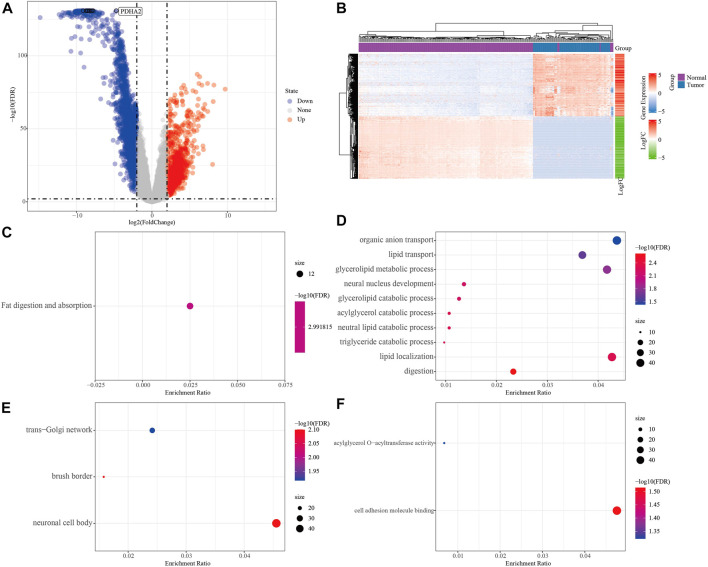
Analysis of PAAD differential genes. **(A)** Volcano plot of differentially expressed genes in expression profile. **(B)** Heat map of differentially expressed genes. **(C)** Results of differential gene KEGG enrichment. **(D)** Results of differential gene GO BP enrichment. **(E)** Differential gene GO CC enrichment results. **(F)** Differential gene GO MF enrichment results, the color from blue to red in CDEF represents FDR from large to small, the size of the dot represents the number of enriched to genes, a larger dot indicates more enriched genes.

To screen the differential genes between normal and KRAS wild-type PAAD samples, we analyzed the differential genes between 171 normal samples and 70 KRAS wild-type tumor samples using the “limma” package, and obtained a total of 2,928 significantly DEGs, of which 2029 were down-regulated and 1,163 were up-regulated in tumors (FDR <0.01, |log2FC| > 2, Figure 2A). A total of 2,928 significantly DEGs were obtained, of which 1,163 were up-regulated and 2029 were down-regulated in tumors (FDR <0.01, |log2FC| > 2, Figure 2A). Then, unsupervised hierarchical clustering of these significantly differentially expressed genes revealed that the differential genes could clearly screen tumor samples from the normal ones (Figure 2B). KEGG study showed that the significant differential genes were mainly related to Fat digestion and absorption (Figure 2C). Biological process (BP) enrichment study demonstrated that the differential genes were largely correlated with Lipid transport, Lipid localization and other pathways; cellular components (CC) showed that the differential genes were associated with neural cell body, trans-Golgi. The results of Molecular Function (MF) showed that the differential genes were related to Cytokine-cytokine receptor interaction, natural killer cell-mediated cytotoxicity, Olfactory transduction, and other such pathways that have been previously reported to be associated with PAAD occurrence Figures 2D–F (Malchiodi and Weiner, 2021; Hu et al., 2022).

**FIGURE 3:**
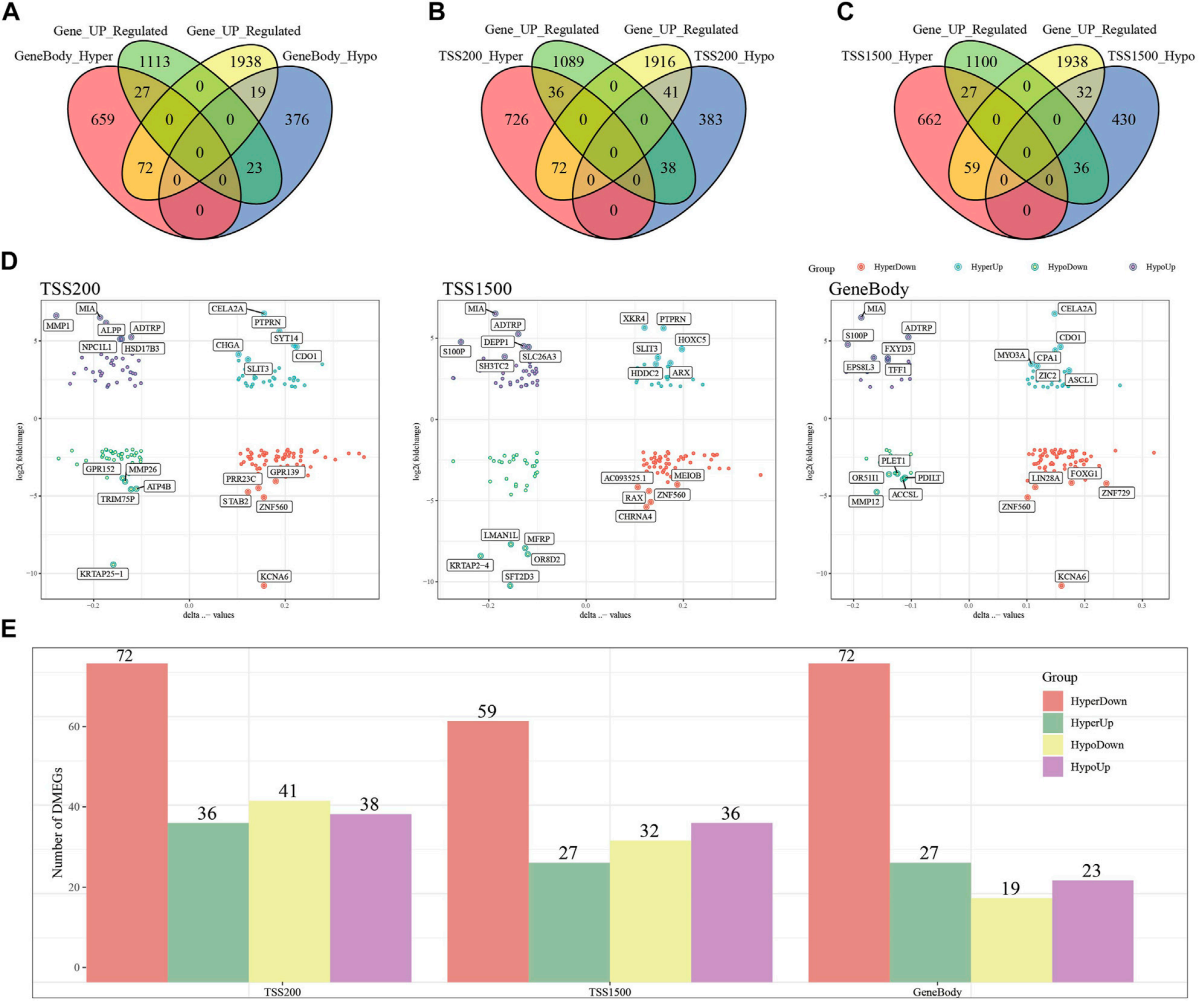
Joint analysis of differentially expressed genes and differentially methylated genes. **(A)** Venn diagram of differentially expressed genes with differentially methylated genes in the GeneBody region. **(B)** Venn diagram of differentially expressed genes with differentially methylated genes in the TSS200 region. **(C)** Venn diagrams of differentially expressed genes versus differentially methylated genes within the TSS1500 region. **(D)** Quadrant plots of differentially expressed genes versus differentially methylated genes within the TSS200, TSS1500, and GeneBody regions. **(E)** Histogram of four regulatory patterns of differentially expressed genes and differentially methylated genes in TSS200, TS1500, and GeneBody.

To search for genes more critical for PAAD occurrence, differentially methylated and expressed genes (DEMGs) were obtained by intersection analysis of DMGs and DEGs. In Gene body, TSS200 and TSS1500, 141, 187 and 154 DEMGs were obtained, respectively (Figures 3A–C). The methylation ploidy and expression difference ploidy of these DMEGs are shown in Figure 3D, and each graph shows the 22 genes with the largest expression difference ploidy. Next, we counted DMEGs in the three regions and identified a total of 318 DMEGs, including 56 in HyperUp, 112 in HyperDown, 69 in HypoUp, and 81 in HypoDwon (Figure 3E).

### 3.3 Analysis of DMEGs genes in PPAD

**FIGURE 4:**
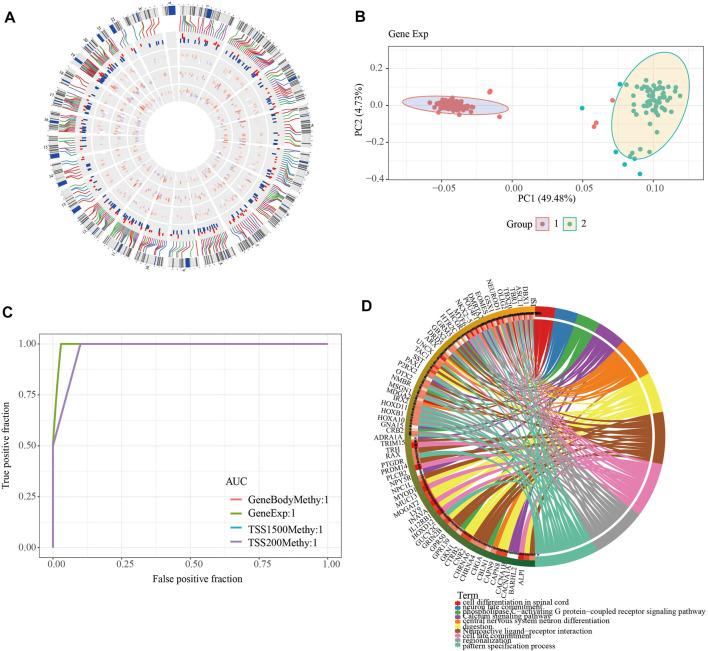
Analysis of DMEGs. **(A)** Distribution of DMEGs on the genome. From inside to outside, there are DMGs in the TSS1500 region, DMGs in the TSS200 region, DMGs in the genebody region, DEGs, and corresponding values. The outermost circle is the corresponding chromosome position. **(B)** PCA analysis could distinguish tumor from normal samples based on the gene expression and methylation of DMEGs. **(C)** ROC curves of tumor and normal samples based on a linear discriminant model using the expression profiles and methylation of DMEGs. **(D)** Results of KEGG and GO enrichment analysis of DMEGs, where different colors represent different pathways and connecting lines represent the existence of association between genes and pathways.

To further investigate the role of DMEGs in PAAD, we first used the “circlize” package to map the distribution of 318 DMEGs on chromosomes, with chromosomes chr11 and chr12 having the largest number of 26 DMEGs, chr10, chr12, chr17, chr16, chr2, chr19, chr3, chr20, chr5, chr4, chr7, chr6, and chr6. Chr17, chr16, chr2, chr19, chr3, chr20, chr5, chr4, chr8, chr7, chr6 chromosomes also possessed more than 10 DMEGs each (Figure 4A). We constructed a linear judgment classification model using the gene expression profiles of DMEGs and methylation data from GeneBody, TSS200 and TSS1500, respectively, to evaluate the difference of DNA methylation patterns and gene expression between PAAD tumors and normal samples, and also performed PCA and ROC analyses. The results of PCA showed that DMEGs could classify PAAD and normal samples effectively (Figure 4B), and the AUC values were all 1, suggesting an excellent performance in classification (Figure 4C). GO and KEGG enrichment analysis showed that DMEGs were mainly associated with cell differentiation in spinal cord, neuron fate commitment, calcium signaling pathway, phospholipase C-activating G protein-coupled receptor signaling pathway, digestion, central nervous system neuron differentiation, neuroactive ligand-receptor interation, cell fate commitment, regionalization, and pattern specification process (Figure 4D).

### 3.4 DMEGs and potential target therapeutic agents

**FIGURE 5:**
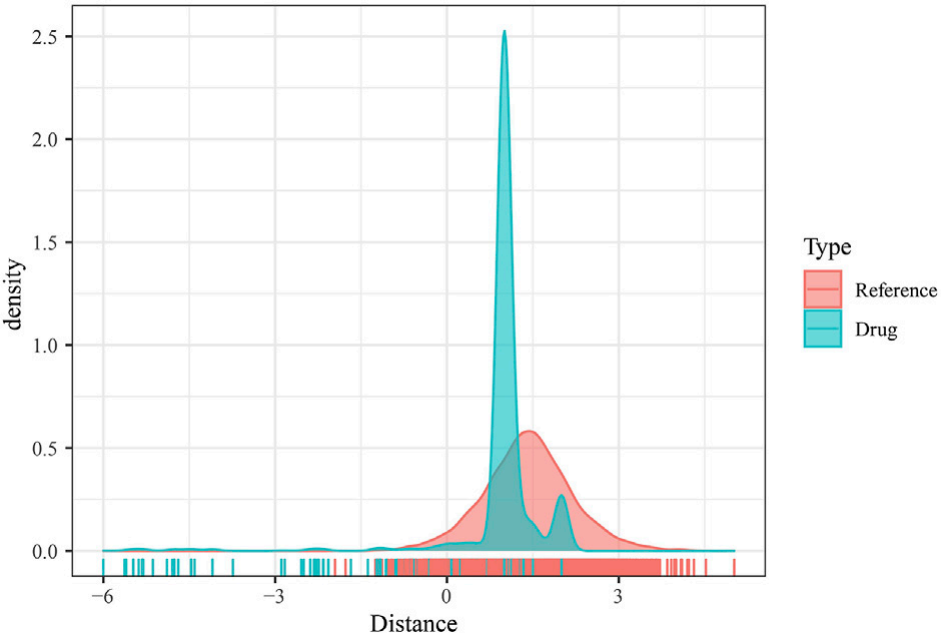
DMEGs and potential target therapeutic drugs. Density fractionation plot of drug to DMEGs gene set distance.

As mentioned previously, DMEGs may be the key genes causing PAAD, and therefore targeting DMEGs is a potential target for the treatment of PAAD. To this end, we calculated the proximity of DMEGs to PAAD according to Formula 1 and converted the observed distances into normalized scores according to Formula 2. We found that either with our randomly selected gene set as a sample or DEMGs as a sample, using the random data acquired for multiple hypothesis testing and selecting drugs with a distance set distributed around 0 to 3 and FDR <0.01, a total of 78 potential target drugs were obtained, and Figure 5 shows the distance density fraction of drugs to DMEGs.

### 3.5 Molecular docking and pharmacokinetic simulation

Currently, the ADRA1A protein does not have any resolved crystal structure. We used the AlphaFold Protein Structure Database website (https://www.alphafold.ebi.ac.uk/) website for ADRA1A homology modeling to obtain the 3D structure of the ADRA1A protein and the Deepsite (https://www.playmolecule.com/deepsite/) website to predict the protein activity of ADRA1A (32). In addition, the Gromacs2019 software package was used to predict potential small molecule compounds, and a total of 10 small molecule compounds were identified by calculating RMSD values, namely DB06201, DB12733, DB00610, DB00450, DB00699, DB06706, DB06711, DB06764 DB00949, and DB08954 (see Table 2).

**TABLE 2:** Molecular docking scores of compounds and proteins and the important interactions generated.

Compound	Compound	Target	Docking score	H-bond interactions
DB06201	Rufinamide	GRM5	−4.874	SER143, SER145, THR168
DB12733	Dipraglurant	GRM5	−4.348	SER145, THR168
DB00610	Metaraminol	ADRA1A	−5.158	MET1, GLU87
DB00450	Droperidol	ADRA1A	−5.819	GLU87
DB00699	Nicergoline	ADRA1A	−2.137	MET1
DB06706	Isometheptene	ADRA1A	−2.752	GLU87
DB06711	Naphazoline	ADRA1A	−5.167	—
DB06764	Tetryzoline	ADRA1A	−5.246	—
**DB00949**	**Felbamate**	**GRIN2B**	−**10.586**	GLU106, SER132
DB08954	Ifenprodil	GRIN2B	−6.821	GLU106, ARG115, ALA135

Taken together, DB0094 (Felbamate) 9 had the highest molecular docking score and therefore had a higher potential to be a potential inhibitor of GRIN2B protein. Compound DB0094 interacted with GRIN2B protein, and the RMSD value of compound DB0094 was relatively stable overall (basically stable at around 3 Å) (Figure 5). The compound was able to produce hydrogen bonding interactions with SER132 and GLU106 of GRIN2B protein, and favorable hydrophobic interactions with ILE111, PRO78, ALA107, PRO177 and ALA135, as well as with TYR109, PHE114 and PHE176. Compound DB00949 (Felbamate) showed a high molecular docking score that many favorable interactions with GRIN2B protein were produced.

**FIGURE 6:**
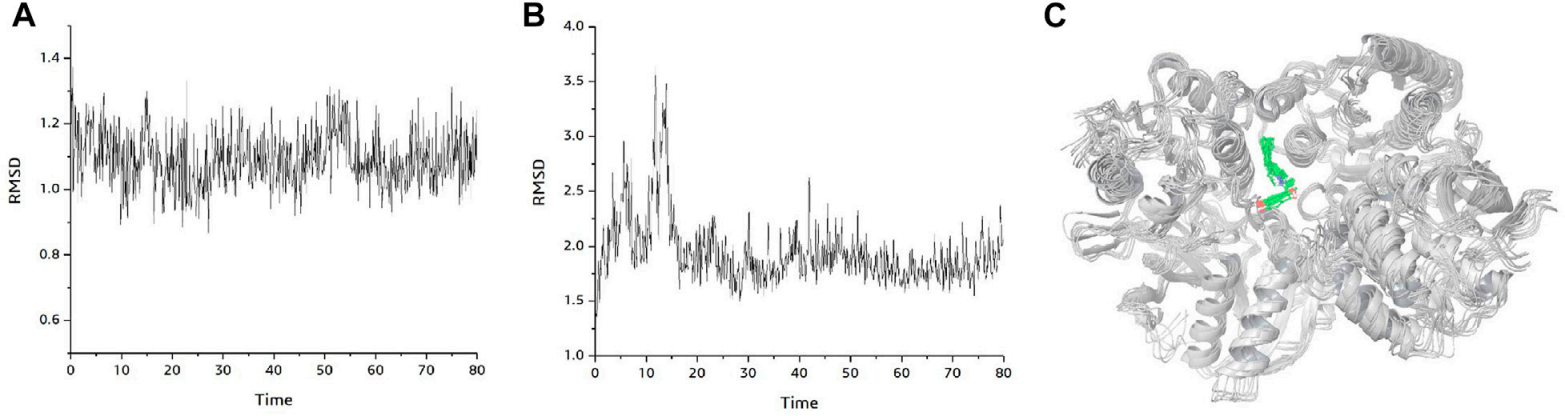
Binding mode plot of GRIN2B protein with compound DB00949(Felbamate). **(A)** RMSD diagram of GRIN2B protein during 80 ns molecular dynamics simulation. **(B)** RMSD values of compound DB00949 (Felbamate) during 80 ns molecular dynamics simulation. **(C)** Plot of the dynamic binding pattern of GRIN2B protein with compound DB00949 (Felbamate) during 80 ns molecular dynamics simulation.

Figure 6A shows the changes of RMSD values of the D-protein backbone of GRIN2B protein bound to compound DB00949 (Felbamate) during the molecular dynamics simulation at 80 ns As can be seen from the figure, the conformation of the GRIN2B protein was very stable during the molecular dynamics simulation at 80 ns, which also indicated to some extent that the protein structure generated based on homology modeling was relatively reasonable (Figure 6B). In addition, Figure 6C gives the RMSD values of the molecular backbone of compound DB00949 (Felbamate) binding to GRIN2B protein during molecular dynamics (MD) simulation of 80 ns The results demonstrated that compound DB00949(Felbamate)’s RMSD value fluctuated relatively large by an obvious increasing trend during the first 20 ns The stability was basically achieved when it reached 20 ns It remained comparatively constant in the subsequent 60 ns Since the molecular docking was semi-flexible in this experiment, it is understandable that the RMSD values of the ligand’s molecular backbone fluctuated moderately in the initial stage of the dynamics simulation. Overall, compound DB00949 (Felbamate) was relatively stable when binding to GRIN2B protein, which further suggested that compound DB00949 (Felbamate) had a high potential to be a potential inhibitor of GRIN2B protein.

### 3.6 Establishment of prognostic gene signature associated with DMEG

**FIGURE 7:**
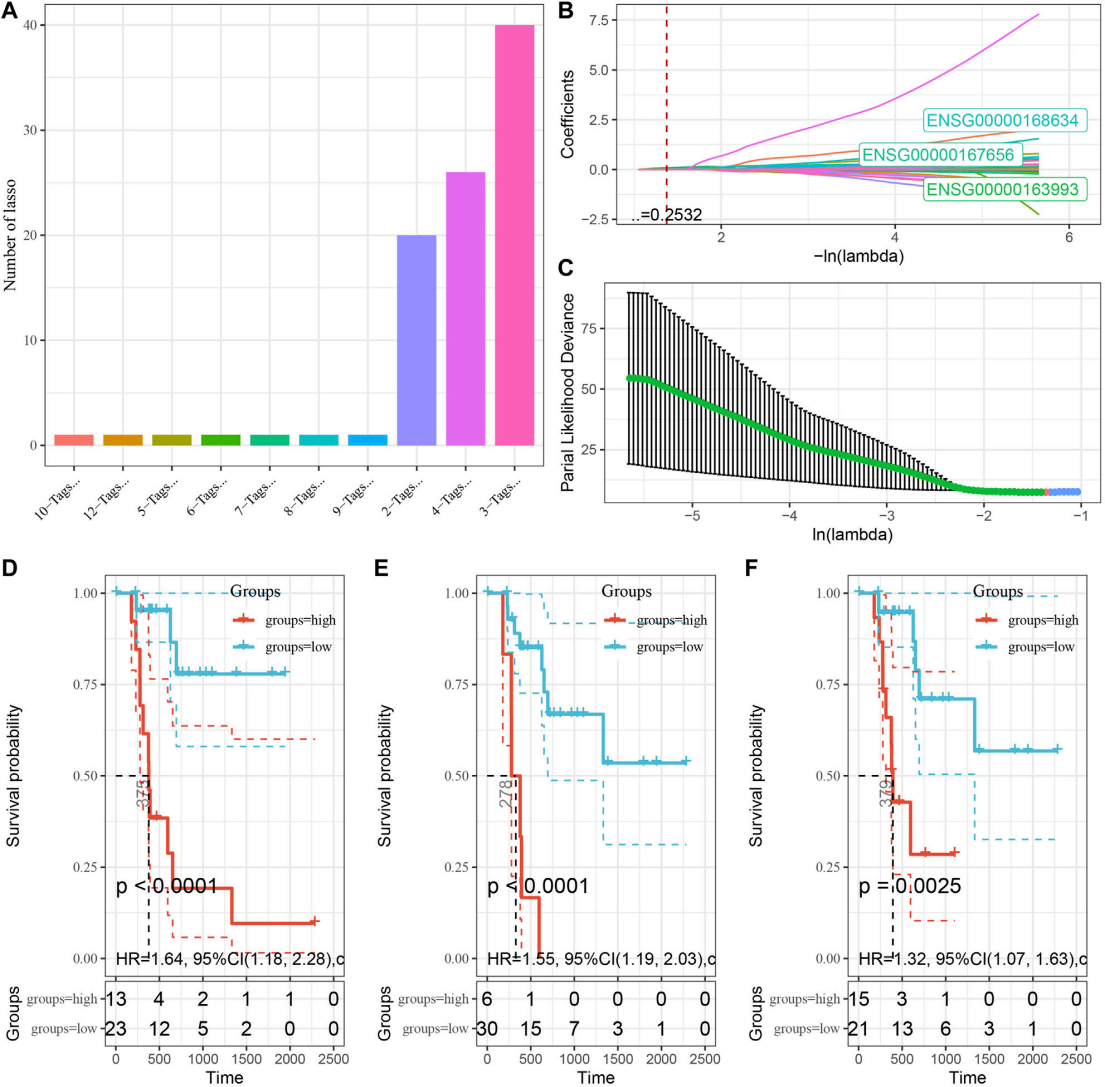
Establishment of prognostic gene signature associated with DMEG. **(A)** Frequency of individual gene combinations for one thousand lasso regressions. **(B)** Coefficient change trajectories of individual genes under different lambda. **(C)** Standard deviation distribution of the models under different lambda. **(D)** Prognostic KM curves of S100P in high and low expression groups. **(E)** Prognostic KM curves of LY6D in high and low expression groups. **(F)** Prognostic KM curves of WFDC13 in high and low expression groups.

To explore the role of DMEG gene expression in PAAD prognosis, we first randomly divided 241 KRAS wild samples into two groups, one as the training set (*n* = 121) and one as the validation set (*n* = 120). We used 10-fold cross-validation to execute 1,000 Lasso regression analysis on the expression and clinical survival data of these 318 DMEGs genes, and we counted the appearances of each probe 100 times (Figure 7A). 3 probes (S100P, LY6D, and WFDC13) appeared the most frequently, and these 3 genes showed the highest frequency with different coefficient of variation trajectories of lambda as Figure 7B, standard deviation distributions of different lambda as Figure 7C. K-M survival curve results indicated that these three genes were able to distinguish more significantly between the two risk groups (Figures 7D–F). Finally, the risk score formula was obtained as follow:

RiskScore=0.44*S100P+0.147*LY6D+0.29*WFDC13

**FIGURE 8:**
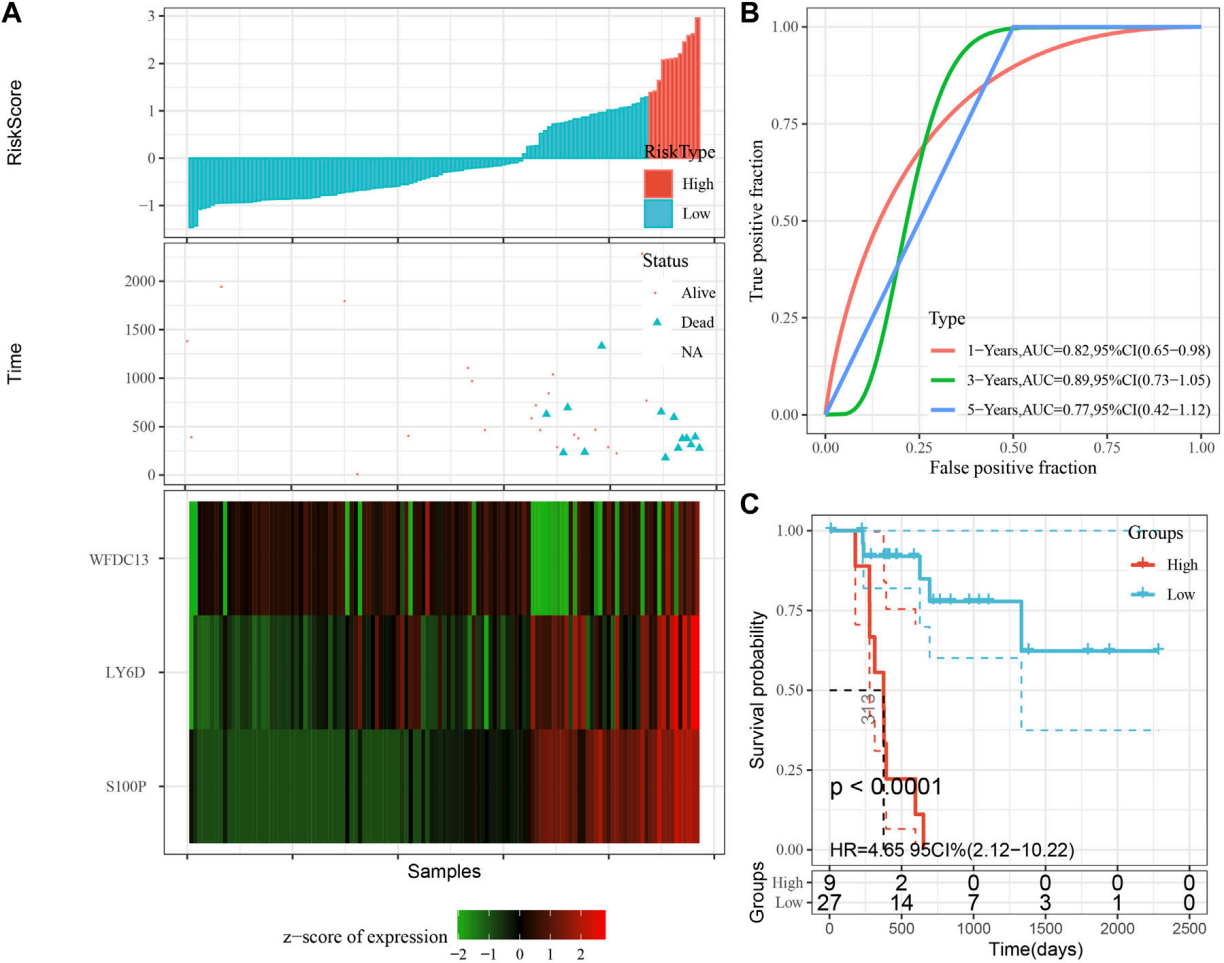
Performance of the prognostic gene signature in training set. **(A)** Risk score, survival time and survival status and expression of the 3 genes in training set. **(B)** ROC curve and AUC of the 3-gene signature classification. **(C)** Distribution of KM survival curves of the 3-gene signature in training set.

According to the expression level of the sample, we calculated the risk score for PAAD samples, and the RiskScore distribution is shown in Figure 8A. From the results of survival analysis, samples with high risk scores showed a significantly worse overall survival (OS) (*p* < 0.001). Then, we used the “timeROC” package to perform ROC analysis for prognostic classification of RiskScore, and the AUCs of predictive classification efficiency were 0.82, 0.89, and 0.77 for one-, three-, and five-year, respectively (Figure 8B), suggesting a good predictive performance. Finally, we performed zscore for Riskscore and determined the cut-off value, divided the sample into high-risk and low-risk groups, and plotted K-M curves. The low-risk group showed significantly better prognosis than that in the high-risk group (Figure 8C, log rank *p* < 0.0001).

### 3.7 Validation of the prognostic gene signature associated with DMEG

**FIGURE 9:**
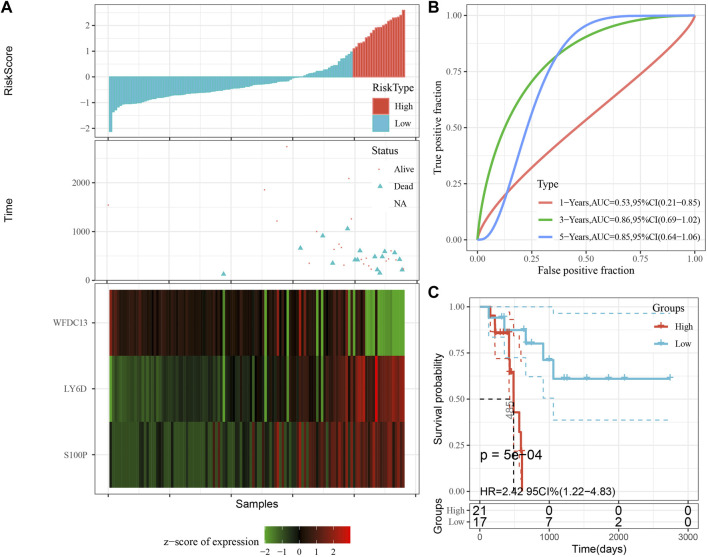
Validation of the prognostic gene signature in validation set. **(A)** Risk score, survival time and survival status and expression of the 3 genes. **(B)** ROC curve and AUC of the 3-gene signature classification. **(C)** Distribution of KM survival curves of the 3-gene signature in the validation set.

**FIGURE 10:**
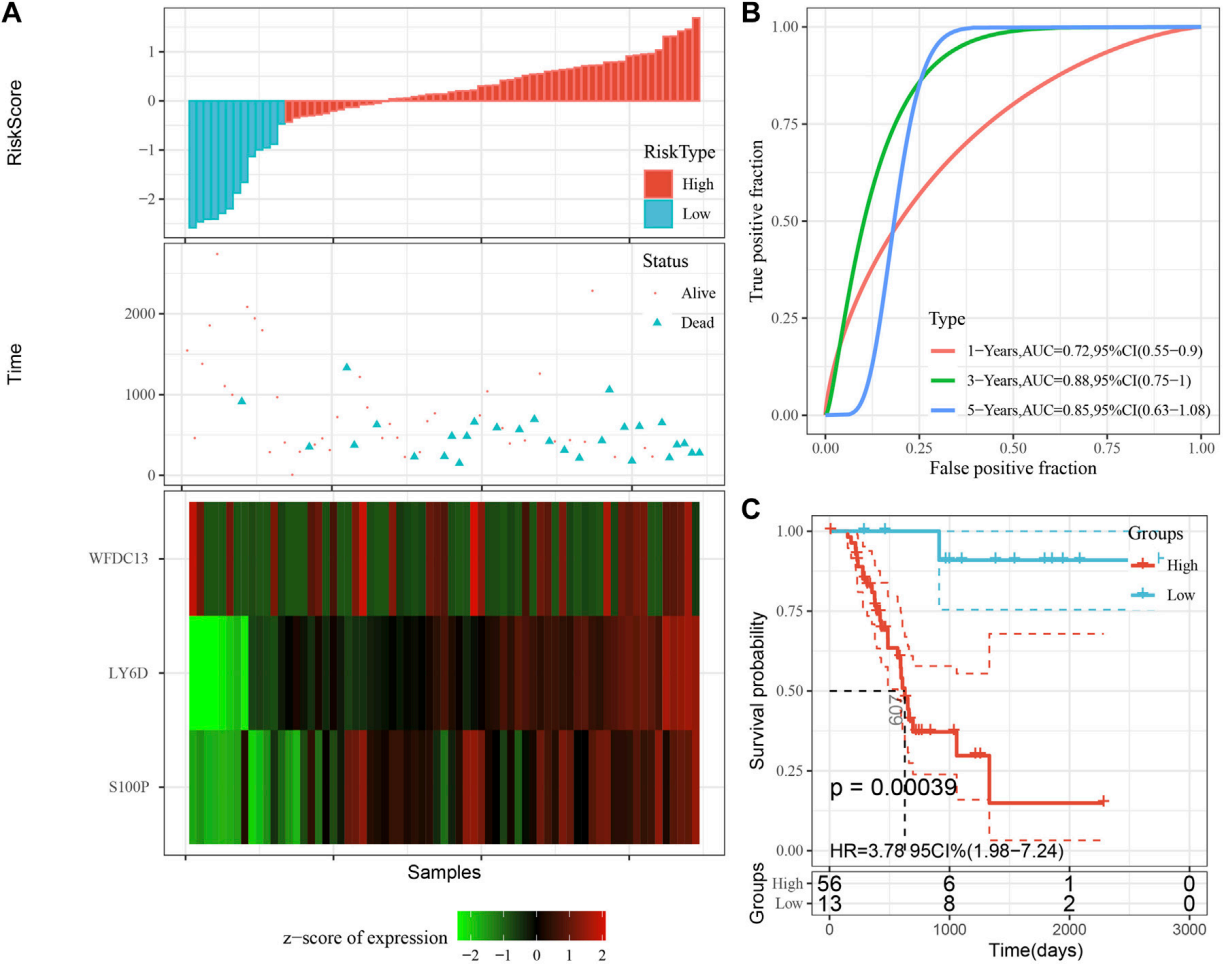
Validation of prognostic gene signatures in KRAS wild-type PAAD samples. **(A)** Risk score, survival time and survival status and expression of the 3 genes in KRAS wild-type samples; **(B)** ROC curves and AUC of the 3-gene signature classification; **(C)** Distribution of KM survival curves of 3-gene signature in TCGA KRAS wild-type samples.

**FIGURE 11:**
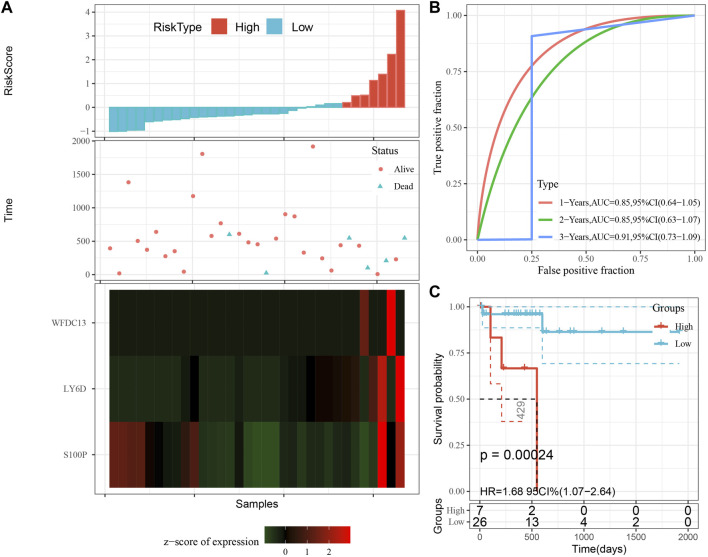
Validation of prognostic gene signatures in external datasets. **(A)** Risk score, survival time vs. survival status and expression of the 3 genes; **(B)** ROC curve and AUC for the 3-gene signature classification; **(C)** Distribution of KM survival curves of 3-gene signature in ICGC-PAAD samples.

The model was validated further by using the same coefficients and model in the training set as in the validation set. The risk score of each sample was calculated using the same method, and the RiskScore distribution is shown in Figure 9A. Similarly, the AUCs of the classification efficiency of the one-year, three-year, and five-year prognostic predictions were 0.53, 0.86, and 0.85, respectively (Figure 9B), and the OS of the high-risk-score samples was significantly worse than that of the low-risk-score samples (Figure 9C, log rank p = 5e x10-4, HR = 2.42). Next, we used the same coefficients and model in the TCGA-PAAD cohort KRAS wild-type group samples as in the training set. We also calculated risk scores for each sample separately based on the expression level of the samples, and the RiskScore distribution is shown in Figure 10A, with AUCs of 0.72, 0.88, and 0.85 for the prognostic predictive classification efficiency at one, three, and 5 years, respectively (Figure 10B). Survival analysis showed that the OS of the high-risk score samples was significantly smaller than that of the low-risk score samples (Figure 10C, log-rank *p* = 0.00039, HR = 3.78). Finally, we performed the same validation in the ICGC-PAAD external data cohort, and the RiskScore distributions for each sample are shown in Figure 11A. The AUCs for prognostic predictive classification efficiency at one, three, and 5 years were 0.85, 0.85, and 0.91, respectively (Figure 11B), and survival analysis showed that the OS of the high-risk score sample was significantly worse than that of the low-risk score sample (Figure 11C, log rank *p* = 0.00024, HR = 1.68).

## 4 Discussion

PAAD as one of the most lethal and aggressive malignancies has a 5-year survival rate of less than 10%, (Jiménez et al., 2017), and is now among the top four leading causes resulting in tumor-associated death (Kleeff et al., 2016). The median age of onset of PAAD is 71 years, and with the aging of the population, its morbidity and mortality will increase rapidly. By 2030, PAAD is estimated as a second cause to tumor mortality (Rahib et al., 2014). The cause of pancreatic cancer is still unclear, and only 5%–10% of pancreatic cancer patients can be attributed to genetic factors (Siegel et al., 2022), although the mutation rate of KRAS reaches 95%, but a single KRAS gene mutation does not lead to the development of pancreatic cancer. Epigenetic alterations are more closely related to environmental and age factors than genetics. Past studies have found that epigenetic alterations occur in the early stages of tumor and are cumulative with tumor development (Nebbioso et al., 2018). In this study, we first DEGs and DMGs in normal samples versus tumor samples without KRAS wild-type based on expression profiling data of pancreatic cancer, and performed functional analysis. Then a classification model was constructed, which can accurately separate normal samples from tumor samples. Finally, we used DMEGs to perform gene-drug interactions on DrugBank to find some potential anti-PAAD drugs, which provides new ideas and potential targets for understanding the role of methylation in PAAD and treating PAAD.

In the early 20th century, Fukushima N and other scholars extensively studied the methylation of different genes in PAAD and its precancerous lesions (intra-epithelial neoplasia (PanIN), and found abnormal methylation of ppENK and p16 (13). Next, it was shown that the incidence of aberrant methylation was 7.3%–7.7% in PanIN-1 patients, 22.7% in PanIN-2 patients, and 46.2% in PanIN-3 patients, a phenomenon that suggests that the incidence of aberrant methylation increases with a more advanced PanIN grade, but the exact mechanism is not clear (Fukushima et al., 2002). Our study, by screening for differentially methylated genes, initially it was found that methylation genes were mediated through Cytokine-cytokine receptor interaction, Natural killer cell-mediated cytotoxicity, Olfactory transduction, and some other pathways leading to the development of PAAD. To further confirm the pathway correlation between PAAD and gene methylation, the intersection of differentially genes and differentially methylated genes was taken and performed enrichment analysis again, and the results demonstrated that methylation led to PAAD by affecting cytokine receptor, NK cell-mediated cytotoxicity pathway.

Illumina human methylation 450 k bead array provides a better technical platform for further study of DNA methylation, therefore, we focused on methylation genes within the three regions of Gene body, TSS1500, and TSS200. A total of 758 hypermethylated genes and 418 demethylated genes were identified within the Gene body region, which was consistent with the incidence of PAAD hypomethylation reported in previous studies, and hypomethylation was mainly associated with cell cycle cycling, cell differentiation, and cell surface antigen/cell adhesion (Pedersen et al., 2011; Schäfer et al., 2021; Zhu et al., 2021). TSS1500 is a functional element belonging to differential methylation and is located between 1.5 kb and 200 bp upstream of the transcription start site. Previous studies identified the TSS1500 region as an oncogenic cofactor variable in lung adenocarcinoma and squamous carcinoma by differential methylation probes, and extensive analysis showed that gene probes outside the TSS1500 region could act as potential pathogenic players by affecting the activity of phosphatidylinositol-3,4,5-trisphosphate (Cao et al., 2022). Our study likewise demonstrated an expression imbalance between hypermethylation and hypomethylation in the TSS1500 region, and by using genes in the TSS1500 region, we were able to construct a classification model to distinguish PAAD from normal tissue, providing a useful tool to identify PAAD. tSS200 also belongs to the transcription factor repressor functional element, and methylation in the TSS200 region is not only related to tumor development, but also involved in the acceleration of epigenetic mutational load and epigenetic age, providing a new perspective for our understanding of the age of DNA methylation (Yan et al., 2020).

In 2005, the European Palliative Care Research Collaborative (EPCRC) network working group screened important clinical markers for survival prediction in patients with end-stage cancer based on decades of clinical evidence and recommended a variety of prognostic tools. On this basis, researchers have successively validated and derived several relevant prediction models according to cancer types, and PAAD prognostic models have emerged, which can be broadly classified into traditional manual prediction and statistical-based bioinformatics modeling, with the latter being the majority at present, but they all share common problems such as small sample size, low specificity, and poor predictive performance (Yuan et al., 2021) (Wang et al., 2021; Zhao et al., 2021). Compared with previous PAAD models, we performed model improvement by combining methylation genes (S100P, LY6D, and WFDC13) with clinical factors in prognostic factors and confirmed the model robustness by external and internal validation. S100P is a member of the S100 protein family containing 2 EF-hand calcium-binding motifs. s100 is localized in the cytoplasm and/or nucleus of a variety of cells and is involved in cell cycle progression and cell differentiation. Meta-analysis showed that S100P is a highly sensitive and highly specific tool for the diagnosis of PAAD (AUC = 0.93) (Hu et al., 2014; Camara et al., 2020). LY6D is mainly involved in lymphoid differentiation and cell surface activity, and the study showed that LY6D is significantly highly expressed in PAAD and is a valid predictor of PAAD, a result consistent with our study (Wang et al., 2020; Xu et al., 2021). WFDC13 belongs to the telomere cluster family of genes, and there are relatively few studies on WFDC13 in PAAD. Our data indicated that WFDC13 was a potential prognostic gene for PAAD and was implicated in the methylation process of PAAD, which provided new ideas for future basic experiments. However, our study was still inadequate and further basic experiments to elucidate the mechanism of the role of this methylation gene in PAAD are required.

There are some limitations in this study. Although the results showed that 3-DMEGs-based signature could distinguish tumor samples and normal samples, the model reliability should be improved with long-term clinical application. Additionally, we downloaded expression profiles and methylation data of PAAD from public databases. Thus, further prospective data should be collected to validate the results. Besides, experimental studies and clinical trials should be performed to verify the results of molecular docking in this study.

## 5 Conclusion

With the gene expression profile data of PAAD, we identified DEGs and DMGs between normal samples and tumor samples with KRAS wild type; the classification model based on DMEGs was able to accurately separate normal samples from tumor samples, and the gene-drug interactions were performed on DrugBank to find some potential anti PAAD drugs.

## Data Availability

The original contributions presented in the study are included in the article/Supplementary Material, further inquiries can be directed to the corresponding authors.
